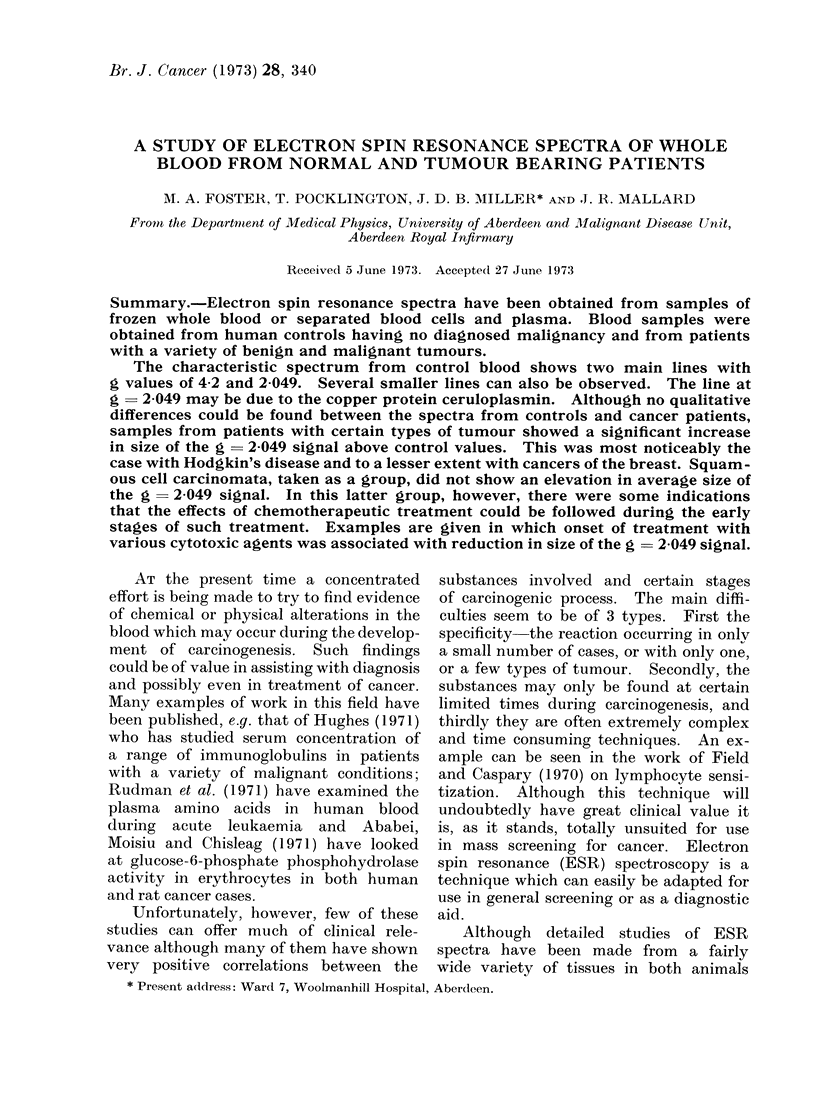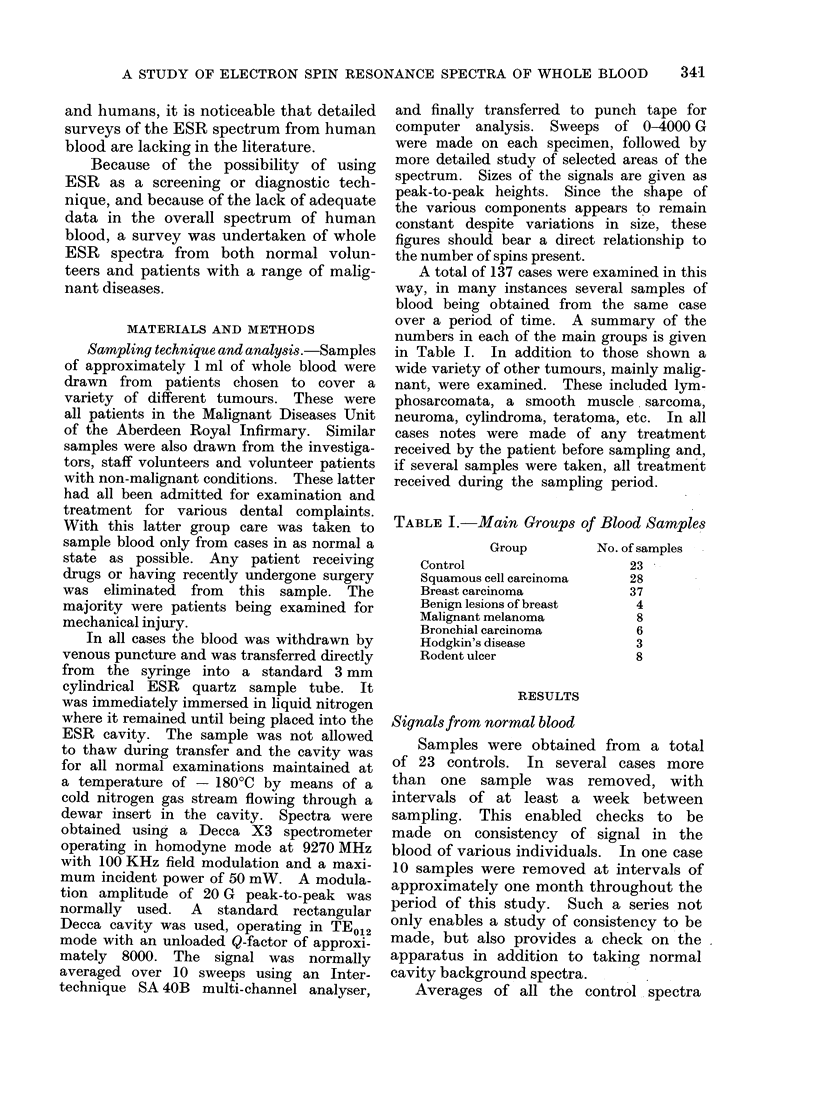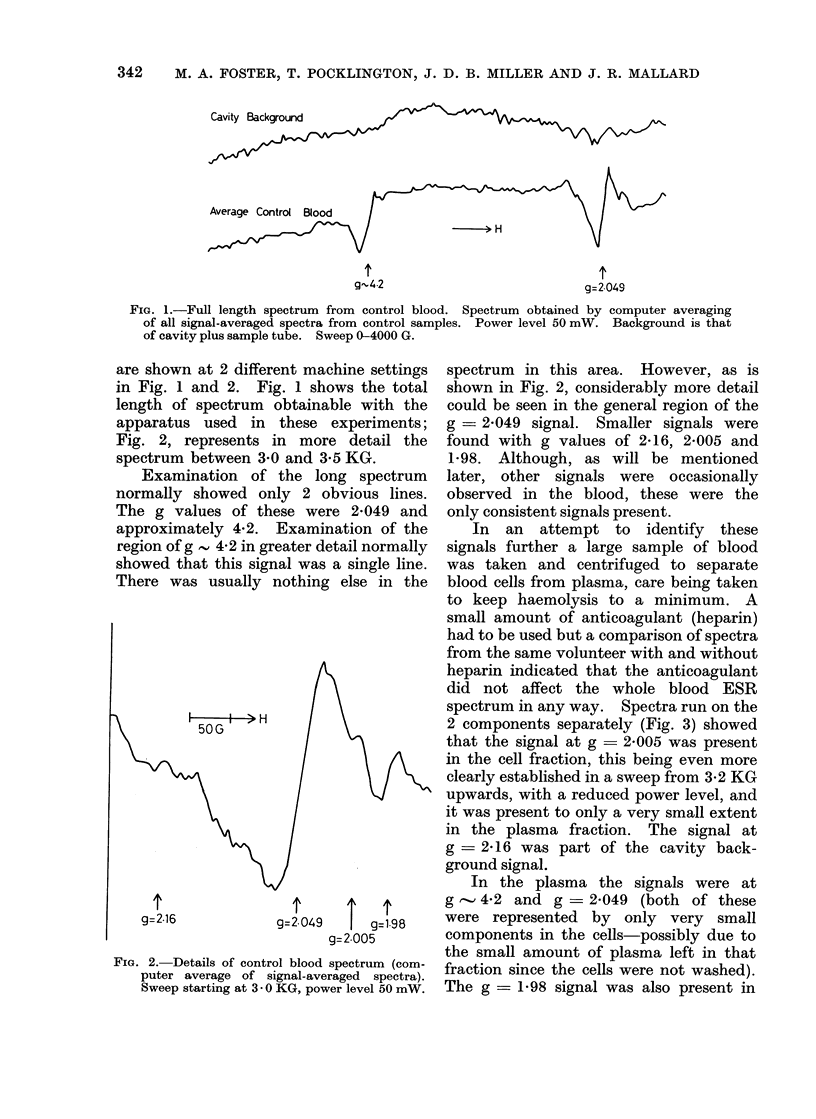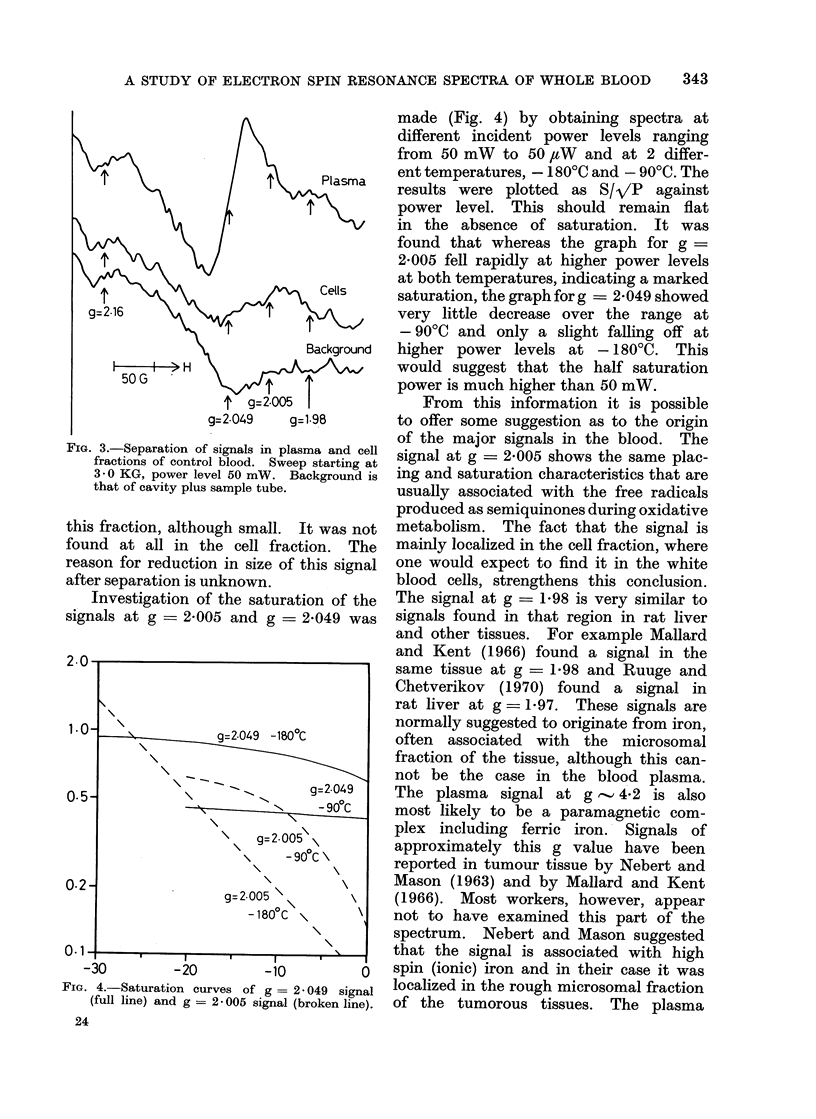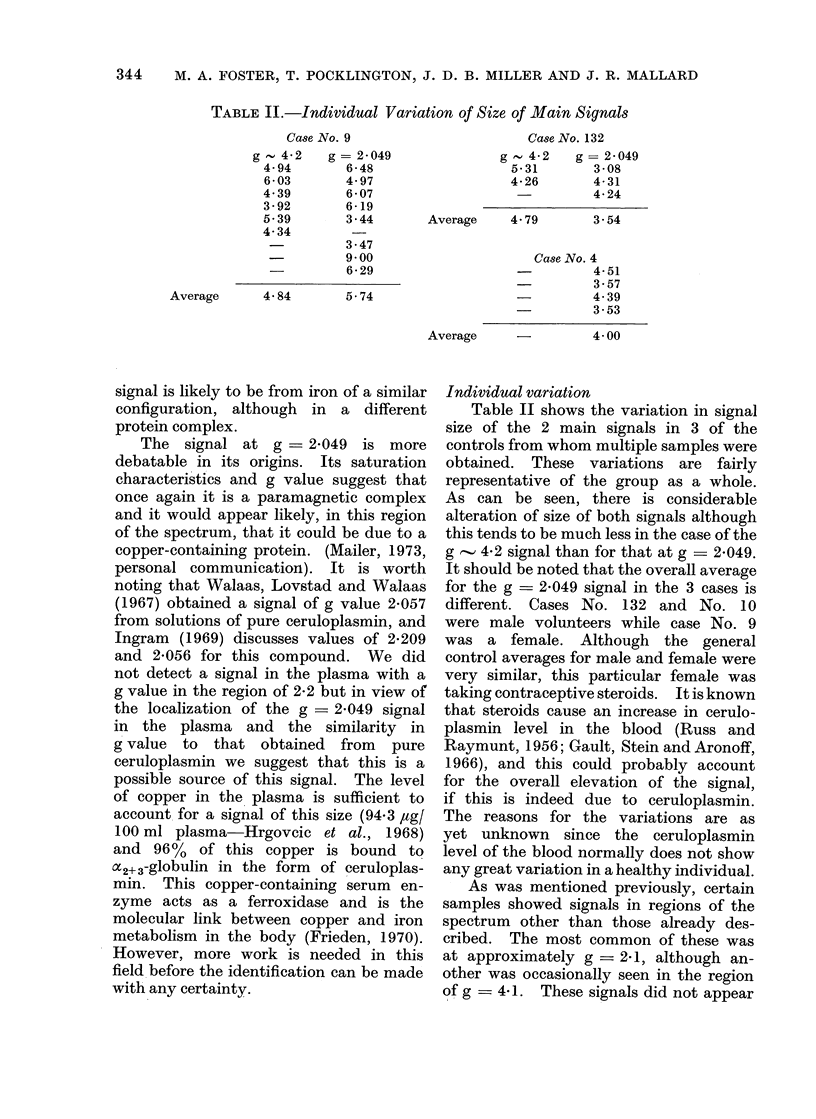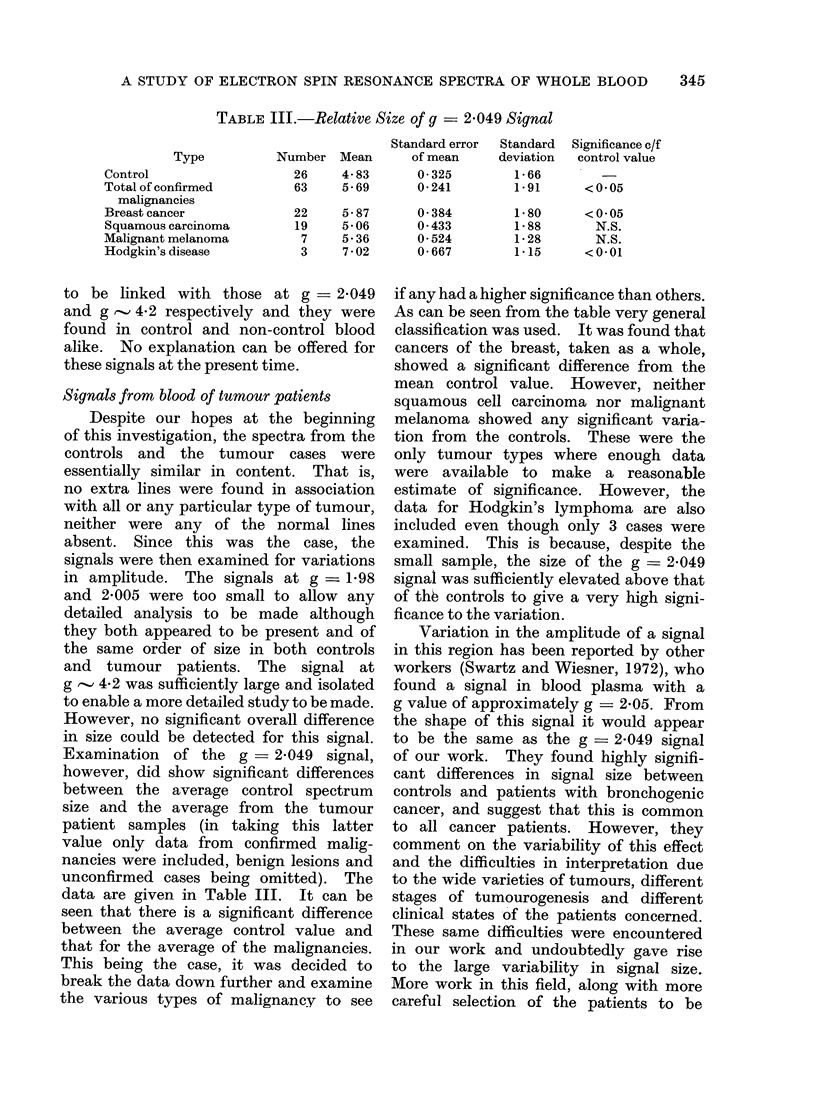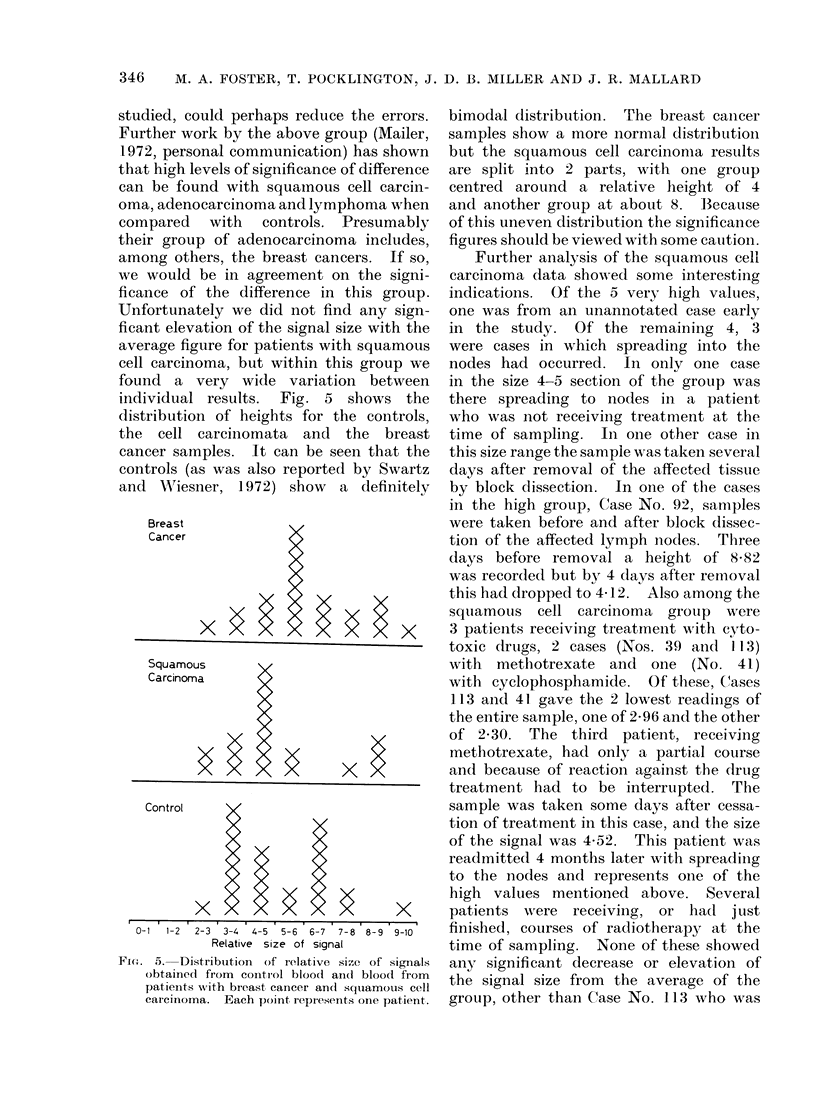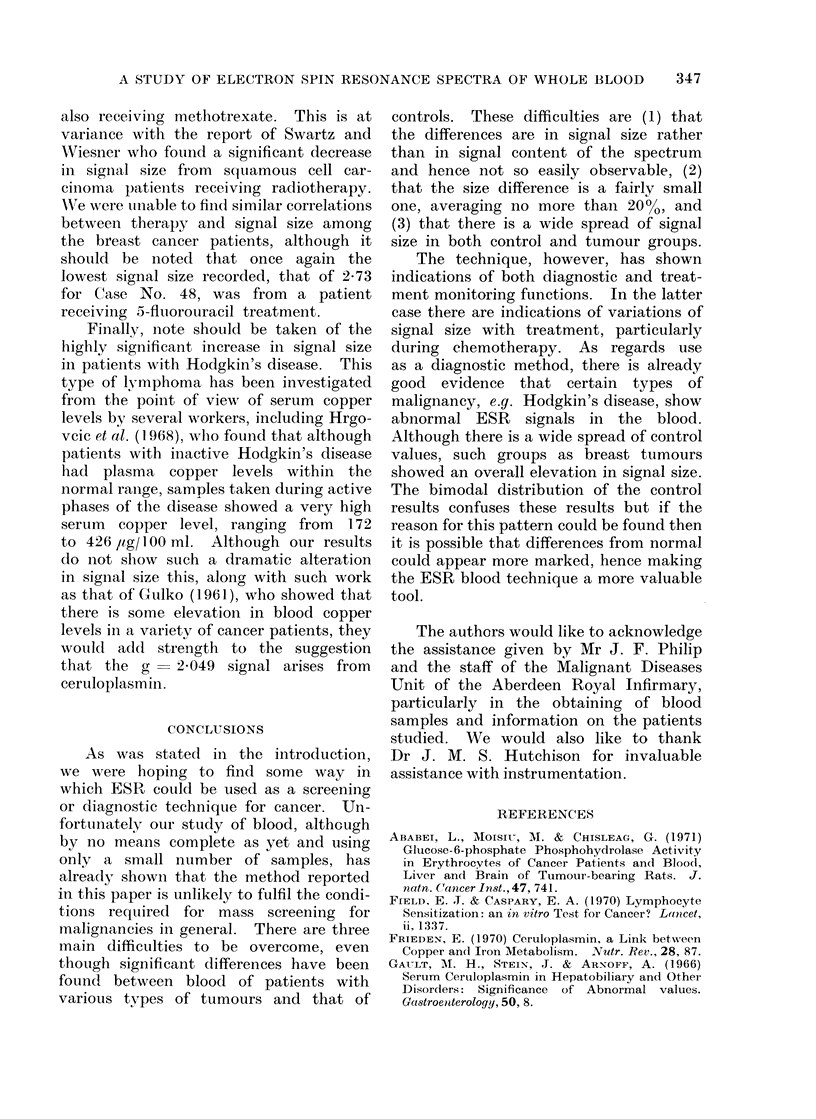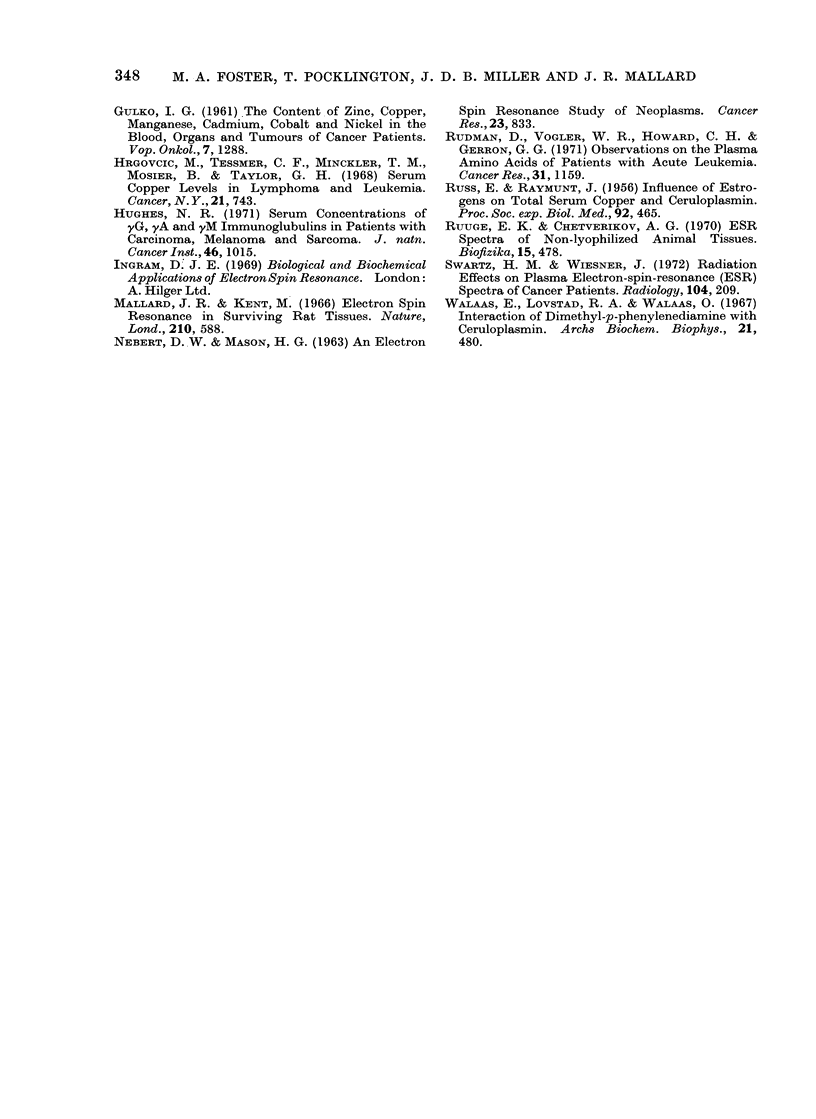# A Study of Electron Spin Resonance Spectra of Whole Blood from Normal and Tumour Bearing Patients

**DOI:** 10.1038/bjc.1973.157

**Published:** 1973-10

**Authors:** M. A. Foster, T. Pocklington, J. D. B. Miller, J. R. Mallard

## Abstract

Electron spin resonance spectra have been obtained from samples of frozen whole blood or separated blood cells and plasma. Blood samples were obtained from human controls having no diagnosed malignancy and from patients with a variety of benign and malignant tumours.

The characteristic spectrum from control blood shows two main lines with g values of 4·2 and 2·049. Several smaller lines can also be observed. The line at g = 2·049 may be due to the copper protein ceruloplasmin. Although no qualitative differences could be found between the spectra from controls and cancer patients, samples from patients with certain types of tumour showed a significant increase in size of the g = 2·049 signal above control values. This was most noticeably the case with Hodgkin's disease and to a lesser extent with cancers of the breast. Squamous cell carcinomata, taken as a group, did not show an elevation in average size of the g = 2·049 signal. In this latter group, however, there were some indications that the effects of chemotherapeutic treatment could be followed during the early stages of such treatment. Examples are given in which onset of treatment with various cytotoxic agents was associated with reduction in size of the g = 2·049 signal.


*Br. J. Cancer* (1973) **28**, 340

### A STUDY OF ELECTRON SPIN RESONANCE SPECTRA OF WHOLE BLOOD FROM NORMAL AND TUMOUR BEARING PATIENTS

M. A. FOSTER, T. POCKLINGTON, J. D. B. MILLER* AND J. R. MALLARD

*From the Department of Medical Physics, University of Aberdeen and Malignant Disease Unit, Aberdeen Royal Infirmary*

Received 5 June 1973. Accepted 27 June 1973

**Summary.—Electron spin resonance spectra have been obtained from samples of frozen whole blood or separated blood cells and plasma. Blood samples were obtained from human controls having no diagnosed malignancy and from patients with a variety of benign and malignant tumours.**

**The characteristic spectrum from control blood shows two main lines with g values of 4·2 and 2·049. Several smaller lines can also be observed. The line at g = 2·049 may be due to the copper protein ceruloplasmin. Although no qualitative differences could be found between the spectra from controls and cancer patients, samples from patients with certain types of tumour showed a significant increase in size of the g = 2·049 signal above control values. This was most noticeably the case with Hodgkin's disease and to a lesser extent with cancers of the breast. Squamous cell carcinomata, taken as a group, did not show an elevation in average size of the g = 2·049 signal. In this latter group, however, there were some indications that the effects of chemotherapeutic treatment could be followed during the early stages of such treatment. Examples are given in which onset of treatment with various cytotoxic agents was associated with reduction in size of the g = 2·049 signal.**

At the present time a concentrated effort is being made to try to find evidence of chemical or physical alterations in the blood which may occur during the development of carcinogenesis. Such findings could be of value in assisting with diagnosis and possibly even in treatment of cancer. Many examples of work in this field have been published, *e.g.* that of Hughes (1971) who has studied serum concentration of a range of immunoglobulins in patients with a variety of malignant conditions; Rudman *et al.* (1971) have examined the plasma amino acids in human blood during acute leukaemia and Ababei, Moisiu and Chisleag (1971) have looked at glucose-6-phosphate phosphohydrolase activity in erythrocytes in both human and rat cancer cases.

Unfortunately, however, few of these studies can offer much of clinical relevance although many of them have shown very positive correlations between the substances involved and certain stages of carcinogenic process. The main difficulties seem to be of 3 types. First the specificity—the reaction occurring in only a small number of cases, or with only one, or a few types of tumour. Secondly, the substances may only be found at certain limited times during carcinogenesis, and thirdly they are often extremely complex and time consuming techniques. An example can be seen in the work of Field and Caspary (1970) on lymphocyte sensitization. Although this technique will undoubtedly have great clinical value it is, as it stands, totally unsuited for use in mass screening for cancer. Electron spin resonance (ESR) spectroscopy is a technique which can easily be adapted for use in general screening or as a diagnostic aid.

Although detailed studies of ESR spectra have been made from a fairly wide variety of tissues in both animals

* Present address: Ward 7, Woolmanhill Hospital, Aberdeen.

and humans, it is noticeable that detailed surveys of the ESR spectrum from human blood are lacking in the literature.

Because of the possibility of using ESR as a screening or diagnostic technique, and because of the lack of adequate data in the overall spectrum of human blood, a survey was undertaken of whole ESR spectra from both normal volunteers and patients with a range of malignant diseases.

### MATERIALS AND METHODS

*Sampling technique and analysis.*—Samples of approximately 1 ml of whole blood were drawn from patients chosen to cover a variety of different tumours. These were all patients in the Malignant Diseases Unit of the Aberdeen Royal Infirmary. Similar samples were also drawn from the investigators, staff volunteers and volunteer patients with non-malignant conditions. These latter had all been admitted for examination and treatment for various dental complaints. With this latter group care was taken to sample blood only from cases in as normal a state as possible. Any patient receiving drugs or having recently undergone surgery was eliminated from this sample. The majority were patients being examined for mechanical injury.

In all cases the blood was withdrawn by venous puncture and was transferred directly from the syringe into a standard 3 mm cylindrical ESR quartz sample tube. It was immediately immersed in liquid nitrogen where it remained until being placed into the ESR cavity. The sample was not allowed to thaw during transfer and the cavity was for all normal examinations maintained at a temperature of − 180°C by means of a cold nitrogen gas stream flowing through a dewar insert in the cavity. Spectra were obtained using a Decca X3 spectrometer operating in homodyne mode at 9270 MHz with 100 KHz field modulation and a maximum incident power of 50 mW. A modulation amplitude of 20 G peak-to-peak was normally used. A standard rectangular Decca cavity was used, operating in $TE_{012}$ mode with an unloaded $Q$-factor of approximately 8000. The signal was normally averaged over 10 sweeps using an Intertechnique SA 40B multi-channel analyser, and finally transferred to punch tape for computer analysis. Sweeps of 0–4000 G were made on each specimen, followed by more detailed study of selected areas of the spectrum. Sizes of the signals are given as peak-to-peak heights. Since the shape of the various components appears to remain constant despite variations in size, these figures should bear a direct relationship to the number of spins present.

A total of 137 cases were examined in this way, in many instances several samples of blood being obtained from the same case over a period of time. A summary of the numbers in each of the main groups is given in Table I. In addition to those shown a wide variety of other tumours, mainly malignant, were examined. These included lymphosarcomata, a smooth muscle sarcoma, neuroma, cylindroma, teratoma, etc. In all cases notes were made of any treatment received by the patient before sampling and, if several samples were taken, all treatment received during the sampling period.

TABLE I.—*Main Groups of Blood Samples*

| Group | No. of samples |
|---|---|
| Control | 23 |
| Squamous cell carcinoma | 28 |
| Breast carcinoma | 37 |
| Benign lesions of breast | 4 |
| Malignant melanoma | 8 |
| Bronchial carcinoma | 6 |
| Hodgkin's disease | 3 |
| Rodent ulcer | 8 |

### RESULTS

### *Signals from normal blood*

Samples were obtained from a total of 23 controls. In several cases more than one sample was removed, with intervals of at least a week between sampling. This enabled checks to be made on consistency of signal in the blood of various individuals. In one case 10 samples were removed at intervals of approximately one month throughout the period of this study. Such a series not only enables a study of consistency to be made, but also provides a check on the apparatus in addition to taking normal cavity background spectra.

Averages of all the control spectra

FIG. 1.—Full length spectrum from control blood. Spectrum obtained by computer averaging of all signal-averaged spectra from control samples. Power level 50 mW. Background is that of cavity plus sample tube. Sweep 0–4000 G.

are shown at 2 different machine settings in Fig. 1 and 2. Fig. 1 shows the total length of spectrum obtainable with the apparatus used in these experiments; Fig. 2, represents in more detail the spectrum between 3·0 and 3·5 KG.

Examination of the long spectrum normally showed only 2 obvious lines. The g values of these were 2·049 and approximately 4·2. Examination of the region of g ~ 4·2 in greater detail normally showed that this signal was a single line. There was usually nothing else in the spectrum in this area. However, as is shown in Fig. 2, considerably more detail could be seen in the general region of the g = 2·049 signal. Smaller signals were found with g values of 2·16, 2·005 and 1·98. Although, as will be mentioned later, other signals were occasionally observed in the blood, these were the only consistent signals present.

FIG. 2.—Details of control blood spectrum (computer average of signal-averaged spectra). Sweep starting at 3·0 KG, power level 50 mW.

In an attempt to identify these signals further a large sample of blood was taken and centrifuged to separate blood cells from plasma, care being taken to keep haemolysis to a minimum. A small amount of anticoagulant (heparin) had to be used but a comparison of spectra from the same volunteer with and without heparin indicated that the anticoagulant did not affect the whole blood ESR spectrum in any way. Spectra run on the 2 components separately (Fig. 3) showed that the signal at g = 2·005 was present in the cell fraction, this being even more clearly established in a sweep from 3·2 KG upwards, with a reduced power level, and it was present to only a very small extent in the plasma fraction. The signal at g = 2·16 was part of the cavity background signal.

In the plasma the signals were at g ~ 4·2 and g = 2·049 (both of these were represented by only very small components in the cells—possibly due to the small amount of plasma left in that fraction since the cells were not washed). The g = 1·98 signal was also present in

FIG. 3.—Separation of signals in plasma and cell fractions of control blood. Sweep starting at 3·0 KG, power level 50 mW. Background is that of cavity plus sample tube.

this fraction, although small. It was not found at all in the cell fraction. The reason for reduction in size of this signal after separation is unknown.

Investigation of the saturation of the signals at g = 2·005 and g = 2·049 was made (Fig. 4) by obtaining spectra at different incident power levels ranging from 50 mW to 50 $\mu$W and at 2 different temperatures, $-180°$C and $-90°$C. The results were plotted as $S/\sqrt{P}$ against power level. This should remain flat in the absence of saturation. It was found that whereas the graph for g = 2·005 fell rapidly at higher power levels at both temperatures, indicating a marked saturation, the graph for g = 2·049 showed very little decrease over the range at $-90°$C and only a slight falling off at higher power levels at $-180°$C. This would suggest that the half saturation power is much higher than 50 mW.

FIG. 4.—Saturation curves of g = 2·049 signal (full line) and g = 2·005 signal (broken line).

From this information it is possible to offer some suggestion as to the origin of the major signals in the blood. The signal at g = 2·005 shows the same placing and saturation characteristics that are usually associated with the free radicals produced as semiquinones during oxidative metabolism. The fact that the signal is mainly localized in the cell fraction, where one would expect to find it in the white blood cells, strengthens this conclusion. The signal at g = 1·98 is very similar to signals found in that region in rat liver and other tissues. For example Mallard and Kent (1966) found a signal in the same tissue at g = 1·98 and Ruuge and Chetverikov (1970) found a signal in rat liver at g = 1·97. These signals are normally suggested to originate from iron, often associated with the microsomal fraction of the tissue, although this cannot be the case in the blood plasma. The plasma signal at g $\sim$ 4·2 is also most likely to be a paramagnetic complex including ferric iron. Signals of approximately this g value have been reported in tumour tissue by Nebert and Mason (1963) and by Mallard and Kent (1966). Most workers, however, appear not to have examined this part of the spectrum. Nebert and Mason suggested that the signal is associated with high spin (ionic) iron and in their case it was localized in the rough microsomal fraction of the tumorous tissues. The plasma

TABLE II.—*Individual Variation of Size of Main Signals*

*Case No.* 9

| | g ~ 4·2 | g = 2·049 |
|---|---|---|
| | 4·94 | 6·48 |
| | 6·03 | 4·97 |
| | 4·39 | 6·07 |
| | 3·92 | 6·19 |
| | 5·39 | 3·44 |
| | 4·34 | — |
| | — | 3·47 |
| | — | 9·00 |
| | — | 6·29 |
| Average | 4·84 | 5·74 |

*Case No.* 132

| | g ~ 4·2 | g = 2·049 |
|---|---|---|
| | 5·31 | 3·08 |
| | 4·26 | 4·31 |
| | — | 4·24 |
| Average | 4·79 | 3·54 |

*Case No.* 4

| | g ~ 4·2 | g = 2·049 |
|---|---|---|
| | — | 4·51 |
| | — | 3·57 |
| | — | 4·39 |
| | — | 3·53 |
| Average | — | 4·00 |

signal is likely to be from iron of a similar configuration, although in a different protein complex.

The signal at g = 2·049 is more debatable in its origins. Its saturation characteristics and g value suggest that once again it is a paramagnetic complex and it would appear likely, in this region of the spectrum, that it could be due to a copper-containing protein. (Mailer, 1973, personal communication). It is worth noting that Walaas, Lovstad and Walaas (1967) obtained a signal of g value 2·057 from solutions of pure ceruloplasmin, and Ingram (1969) discusses values of 2·209 and 2·056 for this compound. We did not detect a signal in the plasma with a g value in the region of 2·2 but in view of the localization of the g = 2·049 signal in the plasma and the similarity in g value to that obtained from pure ceruloplasmin we suggest that this is a possible source of this signal. The level of copper in the plasma is sufficient to account for a signal of this size (94·3 $\mu$g/100 ml plasma—Hrgovcic *et al.*, 1968) and 96% of this copper is bound to $\alpha_{2+3}$-globulin in the form of ceruloplasmin. This copper-containing serum enzyme acts as a ferroxidase and is the molecular link between copper and iron metabolism in the body (Frieden, 1970). However, more work is needed in this field before the identification can be made with any certainty.

*Individual variation*

Table II shows the variation in signal size of the 2 main signals in 3 of the controls from whom multiple samples were obtained. These variations are fairly representative of the group as a whole. As can be seen, there is considerable alteration of size of both signals although this tends to be much less in the case of the g ~ 4·2 signal than for that at g = 2·049. It should be noted that the overall average for the g = 2·049 signal in the 3 cases is different. Cases No. 132 and No. 10 were male volunteers while case No. 9 was a female. Although the general control averages for male and female were very similar, this particular female was taking contraceptive steroids. It is known that steroids cause an increase in ceruloplasmin level in the blood (Russ and Raymunt, 1956; Gault, Stein and Aronoff, 1966), and this could probably account for the overall elevation of the signal, if this is indeed due to ceruloplasmin. The reasons for the variations are as yet unknown since the ceruloplasmin level of the blood normally does not show any great variation in a healthy individual.

As was mentioned previously, certain samples showed signals in regions of the spectrum other than those already described. The most common of these was at approximately g = 2·1, although another was occasionally seen in the region of g = 4·1. These signals did not appear

TABLE III.—*Relative Size of $g = 2{\cdot}049$ Signal*

| Type | Number | Mean | Standard error of mean | Standard deviation | Significance c/f control value |
|---|---|---|---|---|---|
| Control | 26 | 4·83 | 0·325 | 1·66 | — |
| Total of confirmed malignancies | 63 | 5·69 | 0·241 | 1·91 | <0·05 |
| Breast cancer | 22 | 5·87 | 0·384 | 1·80 | <0·05 |
| Squamous carcinoma | 19 | 5·06 | 0·433 | 1·88 | N.S. |
| Malignant melanoma | 7 | 5·36 | 0·524 | 1·28 | N.S. |
| Hodgkin's disease | 3 | 7·02 | 0·667 | 1·15 | <0·01 |

to be linked with those at $g = 2{\cdot}049$ and $g \sim 4{\cdot}2$ respectively and they were found in control and non-control blood alike. No explanation can be offered for these signals at the present time.

*Signals from blood of tumour patients*

Despite our hopes at the beginning of this investigation, the spectra from the controls and the tumour cases were essentially similar in content. That is, no extra lines were found in association with all or any particular type of tumour, neither were any of the normal lines absent. Since this was the case, the signals were then examined for variations in amplitude. The signals at $g = 1{\cdot}98$ and $2{\cdot}005$ were too small to allow any detailed analysis to be made although they both appeared to be present and of the same order of size in both controls and tumour patients. The signal at $g \sim 4{\cdot}2$ was sufficiently large and isolated to enable a more detailed study to be made. However, no significant overall difference in size could be detected for this signal. Examination of the $g = 2{\cdot}049$ signal, however, did show significant differences between the average control spectrum size and the average from the tumour patient samples (in taking this latter value only data from confirmed malignancies were included, benign lesions and unconfirmed cases being omitted). The data are given in Table III. It can be seen that there is a significant difference between the average control value and that for the average of the malignancies. This being the case, it was decided to break the data down further and examine the various types of malignancy to see if any had a higher significance than others. As can be seen from the table very general classification was used. It was found that cancers of the breast, taken as a whole, showed a significant difference from the mean control value. However, neither squamous cell carcinoma nor malignant melanoma showed any significant variation from the controls. These were the only tumour types where enough data were available to make a reasonable estimate of significance. However, the data for Hodgkin's lymphoma are also included even though only 3 cases were examined. This is because, despite the small sample, the size of the $g = 2{\cdot}049$ signal was sufficiently elevated above that of the controls to give a very high significance to the variation.

Variation in the amplitude of a signal in this region has been reported by other workers (Swartz and Wiesner, 1972), who found a signal in blood plasma with a g value of approximately $g = 2{\cdot}05$. From the shape of this signal it would appear to be the same as the $g = 2{\cdot}049$ signal of our work. They found highly significant differences in signal size between controls and patients with bronchogenic cancer, and suggest that this is common to all cancer patients. However, they comment on the variability of this effect and the difficulties in interpretation due to the wide varieties of tumours, different stages of tumourogenesis and different clinical states of the patients concerned. These same difficulties were encountered in our work and undoubtedly gave rise to the large variability in signal size. More work in this field, along with more careful selection of the patients to be studied, could perhaps reduce the errors. Further work by the above group (Mailer, 1972, personal communication) has shown that high levels of significance of difference can be found with squamous cell carcinoma, adenocarcinoma and lymphoma when compared with controls. Presumably their group of adenocarcinoma includes, among others, the breast cancers. If so, we would be in agreement on the significance of the difference in this group. Unfortunately we did not find any signficant elevation of the signal size with the average figure for patients with squamous cell carcinoma, but within this group we found a very wide variation between individual results. Fig. 5 shows the distribution of heights for the controls, the cell carcinomata and the breast cancer samples. It can be seen that the controls (as was also reported by Swartz and Wiesner, 1972) show a definitely bimodal distribution. The breast cancer samples show a more normal distribution but the squamous cell carcinoma results are split into 2 parts, with one group centred around a relative height of 4 and another group at about 8. Because of this uneven distribution the significance figures should be viewed with some caution.

FIG. 5.—Distribution of relative size of signals obtained from control blood and blood from patients with breast cancer and squamous cell carcinoma. Each point represents one patient.

Further analysis of the squamous cell carcinoma data showed some interesting indications. Of the 5 very high values, one was from an unannotated case early in the study. Of the remaining 4, 3 were cases in which spreading into the nodes had occurred. In only one case in the size 4–5 section of the group was there spreading to nodes in a patient who was not receiving treatment at the time of sampling. In one other case in this size range the sample was taken several days after removal of the affected tissue by block dissection. In one of the cases in the high group, Case No. 92, samples were taken before and after block dissection of the affected lymph nodes. Three days before removal a height of 8·82 was recorded but by 4 days after removal this had dropped to 4·12. Also among the squamous cell carcinoma group were 3 patients receiving treatment with cytotoxic drugs, 2 cases (Nos. 39 and 113) with methotrexate and one (No. 41) with cyclophosphamide. Of these, Cases 113 and 41 gave the 2 lowest readings of the entire sample, one of 2·96 and the other of 2·30. The third patient, receiving methotrexate, had only a partial course and because of reaction against the drug treatment had to be interrupted. The sample was taken some days after cessation of treatment in this case, and the size of the signal was 4·52. This patient was readmitted 4 months later with spreading to the nodes and represents one of the high values mentioned above. Several patients were receiving, or had just finished, courses of radiotherapy at the time of sampling. None of these showed any significant decrease or elevation of the signal size from the average of the group, other than Case No. 113 who was also receiving methotrexate. This is at variance with the report of Swartz and Wiesner who found a significant decrease in signal size from squamous cell carcinoma patients receiving radiotherapy. We were unable to find similar correlations between therapy and signal size among the breast cancer patients, although it should be noted that once again the lowest signal size recorded, that of 2·73 for Case No. 48, was from a patient receiving 5-fluorouracil treatment.

Finally, note should be taken of the highly significant increase in signal size in patients with Hodgkin's disease. This type of lymphoma has been investigated from the point of view of serum copper levels by several workers, including Hrgovcic *et al.* (1968), who found that although patients with inactive Hodgkin's disease had plasma copper levels within the normal range, samples taken during active phases of the disease showed a very high serum copper level, ranging from 172 to 426 $\mu$g/100 ml. Although our results do not show such a dramatic alteration in signal size this, along with such work as that of Gulko (1961), who showed that there is some elevation in blood copper levels in a variety of cancer patients, they would add strength to the suggestion that the $g = 2{\cdot}049$ signal arises from ceruloplasmin.

### CONCLUSIONS

As was stated in the introduction, we were hoping to find some way in which ESR could be used as a screening or diagnostic technique for cancer. Unfortunately our study of blood, although by no means complete as yet and using only a small number of samples, has already shown that the method reported in this paper is unlikely to fulfil the conditions required for mass screening for malignancies in general. There are three main difficulties to be overcome, even though significant differences have been found between blood of patients with various types of tumours and that of controls. These difficulties are (1) that the differences are in signal size rather than in signal content of the spectrum and hence not so easily observable, (2) that the size difference is a fairly small one, averaging no more than 20%, and (3) that there is a wide spread of signal size in both control and tumour groups.

The technique, however, has shown indications of both diagnostic and treatment monitoring functions. In the latter case there are indications of variations of signal size with treatment, particularly during chemotherapy. As regards use as a diagnostic method, there is already good evidence that certain types of malignancy, *e.g.* Hodgkin's disease, show abnormal ESR signals in the blood. Although there is a wide spread of control values, such groups as breast tumours showed an overall elevation in signal size. The bimodal distribution of the control results confuses these results but if the reason for this pattern could be found then it is possible that differences from normal could appear more marked, hence making the ESR blood technique a more valuable tool.

The authors would like to acknowledge the assistance given by Mr J. F. Philip and the staff of the Malignant Diseases Unit of the Aberdeen Royal Infirmary, particularly in the obtaining of blood samples and information on the patients studied. We would also like to thank Dr J. M. S. Hutchison for invaluable assistance with instrumentation.

### REFERENCES

Ababei, L., Moisiu, M. & Chisleag, G. (1971) Glucose-6-phosphate Phosphohydrolase Activity in Erythrocytes of Cancer Patients and Blood, Liver and Brain of Tumour-bearing Rats. *J. natn. Cancer Inst.*, **47**, 741.

Field, E. J. & Caspary, E. A. (1970) Lymphocyte Sensitization: an *in vitro* Test for Cancer? *Lancet*, ii, 1337.

Frieden, E. (1970) Ceruloplasmin, a Link between Copper and Iron Metabolism. *Nutr. Rev.*, **28**, 87.

Gault, M. H., Stein, J. & Arnoff, A. (1966) Serum Ceruloplasmin in Hepatobiliary and Other Disorders: Significance of Abnormal values. *Gastroenterology*, **50**, 8.

GULKO, I. G. (1961) The Content of Zinc, Copper, Manganese, Cadmium, Cobalt and Nickel in the Blood, Organs and Tumours of Cancer Patients. *Vop. Onkol.*, **7**, 1288.

HRGOVCIC, M., TESSMER, C. F., MINCKLER, T. M., MOSIER, B. & TAYLOR, G. H. (1968) Serum Copper Levels in Lymphoma and Leukemia. *Cancer, N.Y.*, **21**, 743.

HUGHES, N. R. (1971) Serum Concentrations of $\gamma$G, $\gamma$A and $\gamma$M Immunoglubulins in Patients with Carcinoma, Melanoma and Sarcoma. *J. natn. Cancer Inst.*, **46**, 1015.

INGRAM, D. J. E. (1969) *Biological and Biochemical Applications of Electron Spin Resonance*. London: A. Hilger Ltd.

MALLARD, J. R. & KENT, M. (1966) Electron Spin Resonance in Surviving Rat Tissues. *Nature, Lond.*, **210**, 588.

NEBERT, D. W. & MASON, H. G. (1963) An Electron Spin Resonance Study of Neoplasms. *Cancer Res.*, **23**, 833.

RUDMAN, D., VOGLER, W. R., HOWARD, C. H. & GERRON, G. G. (1971) Observations on the Plasma Amino Acids of Patients with Acute Leukemia. *Cancer Res.*, **31**, 1159.

RUSS, E. & RAYMUNT, J. (1956) Influence of Estrogens on Total Serum Copper and Ceruloplasmin. *Proc. Soc. exp. Biol. Med.*, **92**, 465.

RUUGE, E. K. & CHETVERIKOV, A. G. (1970) ESR Spectra of Non-lyophilized Animal Tissues. *Biofizika*, **15**, 478.

SWARTZ, H. M. & WIESNER, J. (1972) Radiation Effects on Plasma Electron-spin-resonance (ESR) Spectra of Cancer Patients. *Radiology*, **104**, 209.

WALAAS, E., LOVSTAD, R. A. & WALAAS, O. (1967) Interaction of Dimethyl-*p*-phenylenediamine with Ceruloplasmin. *Archs Biochem. Biophys.*, **21**, 480.